# Parental perception of antibiotic use in children at home: a cross-sectional study

**DOI:** 10.1590/0034-7167-2025-0030

**Published:** 2025-11-17

**Authors:** Glaubervania Alves Lima, Francisca Elisângela Teixeira Lima, Luis Angel Cendejas Medina, Sara Emilly Lima Sombra, Fernanda Jorge Magalhães, Sabrina de Souza Gurgel Florencio, Maria Gabriela Miranda Fontenele, Lorena Pinheiro Barbosa

**Affiliations:** I Universidade Federal do Ceará. Fortaleza, Ceará, Brazil; II Universidade de São Paulo. Ribeirão Preto, São Paulo, Brazil; III Universidade Estadual do Ceará. Fortaleza, Ceará, Brazil

**Keywords:** Child Health, Respiratory Tract Infections, Anti-Bacterial Agents, Nursing, Perception., Salud Infantil, Infecciones del Sistema Respiratorio, Antibacterianos, Enfermería, Percepción.

## Abstract

**Objectives:**

to assess parental perception of the factors influencing their children’s antibiotic use at home and to determine the association with sociodemographic characteristics.

**Methods:**

a descriptive, cross-sectional study was conducted with 209 parents of children under 12 years of age who used antibiotics at home. A sociodemographic and clinical characterization instrument and the Parental Perception on Antibiotics Scale were administered. The Mann-Whitney, Kruskal-Wallis, and Spearman tests were used for data analysis.

**Results:**

most participants were mothers (90.9%). Parents with elementary education and income below the minimum wage had higher medians in the scale factors 1- Knowledge and beliefs, 2- Behaviors, and 3- Adherence, indicating less understanding of the correct use of antibiotics.

**Conclusions:**

parental perception of the factors that influence antibiotic administration to children at home is directly associated with the correct use of medication and sociodemographic factors.

## RESUMEN

**Objetivos:**

evaluar la percepción de los padres sobre los factores que influyen en el uso de antibióticos en el hogar de sus hijos y determinar su asociación con las características sociodemográficas.

**Métodos:**

se realizó un estudio descriptivo y transversal con 209 padres de niños menores de 12 años que usaban antibióticos en el hogar. Se administró un instrumento de caracterización sociodemográfica y clínica, así como la Escala de Percepción Parental de Antibióticos. Se utilizaron las pruebas de Mann-Whitney, Kruskal-Wallis y Spearman para el análisis de datos.

**Resultados:**

la mayoría de los participantes eran madres (90,9%). Los padres con educación primaria e ingresos inferiores al salario mínimo presentaron medianas más altas en los factores de la escala 1- Conocimientos y creencias, 2- Comportamientos, y 3- Adherencia, lo que indica una menor comprensión del uso correcto de los antibióticos.

**Conclusiones:**

la percepción de los padres sobre los factores que influyen en la administración de antibióticos a los niños en el hogar se asocia directamente con el uso correcto de la medicación y los factores sociodemográficos.

## INTRODUCTION

Respiratory and digestive infections, such as flu, colds, pneumonia, ear infections and gastroenteritis, are common in childhood^(1,2)^. Children’s entry into school settings increases their susceptibility to these infections, making them more exposed to the use of medications, including antibiotics^(3,4)^.

Although antibiotics are essential for treating bacterial infections, their inappropriate or excessive use can have serious consequences. Associated problems include the development of bacterial resistance, the increase in chronic diseases, and the impact on healthcare costs^(5-7)^. These effects are even more worrying in children, negatively influencing their growth and long-term health^(2)^. Therefore, the unnecessary and inappropriate use of antibiotics is considered one of the greatest global public health challenges^(8,9)^.

Several factors contribute to antibiotic misuse, including inappropriate medical prescriptions, lack of knowledge, and misguided attitudes of parents or guardians^(10)^. Self-medication and the purchase of drugs without a prescription remain common practices, especially in contexts where access to healthcare services is limited^(11)^. Furthermore, people’s negative perception of doctor-patient interactions, dissatisfaction with care, and previous successful experiences with antibiotic use can reinforce inappropriate behavior^(12)^. However, this phenomenon is also strongly linked to structural public health problems, such as limited access to medical services and a shortage of professionals, especially in more vulnerable areas. Furthermore, socioeconomic factors, such as low education levels, insufficient income, and limited access to reliable information on the rational use of medications, contribute to inadequate practices in childhood disease management^(4,10)^.

Therefore, given these findings and the gap in Brazilian literature regarding caregivers’ understanding of risks associated with administering antibiotics to their children, the concern that informed this study arose. Given this scenario, understanding how parents perceive their children’s antibiotic use is crucial to identifying the factors that influence inappropriate practices and supporting the development of educational strategies focused on home antibiotic therapy management. Such interventions can promote more effective communication between caregivers and healthcare professionals, establish appropriate therapeutic regimens, and prevent treatment failures that compromise children’s health^(13,14)^.

Thus, this study contributes to clinical and academic practice by providing support for the development of technologies and educational strategies more aligned with families’ needs and realities. By adopting caregivers’ perspective, the research deepens the analysis of the factors that motivate inappropriate practices in the home use of antibiotics, contributing to the promotion of policies and actions that favor the rational use of these medications and safety in childcare.

## OBJECTIVES

To assess parental perception of the factors that influence their children’s use of antibiotics at home and to verify the association with sociodemographic characteristics.

## METHODS

### Ethical aspects

The study was submitted to and approved by the Research Ethics Committee, in accordance with the ethical precepts of Resolution 466/2012 of the Brazilian National Health Council.

### Study design

This is a cross-sectional, descriptive study, carried out in the city of Fortaleza, Ceará, from September to November 2022. The manuscript preparation followed Strengthening the Reporting of Observational Studies in Epidemiology guidelines^(15)^.

### Population, sample and selection criteria

To calculate the sample size, a proportion (P) of 50% of parents of children who had ever used antibiotics at home was estimated, with a 95% confidence level (z = 1.96) and an absolute sampling error of 5%. These parameters were applied to the formula for finite populations, considering an N of 450 parents registered in the two participating schools, resulting in a sample size (n) of 207 participants:


$$n=\frac{N\times Z^{2}\times p(1-p)}{e^{2}(N-1)+Z^{2}\times p(1-p)}$$

However, during data collection, 209 parents responded to the instrument, making up the final study sample. They met the following inclusion criteria: being a parent of a child under 12 years of age who had used antibiotics at home in the six months prior to data collection and having a smartphone with internet access. Individuals with cognitive impairments or who did not respond to three contact attempts were excluded.

Data collection was conducted at two preschools and elementary schools in Fortaleza, Ceará. To broaden sample size and include parents whose children were not enrolled in these schools but had used antibiotics at home, a snowball sampling strategy was adopted, in which participants nominated other parents with a compatible profile to participate in the study.

Administratively, Fortaleza is divided into 12 Regional Executive Departments (RED). The selected schools are located in a neighborhood belonging to RED IX, considered one of the city’s most vulnerable areas. This area presents significant socioeconomic challenges, such as low Human Development Indexes and high population density. These educational institutions were chosen intentionally, justifying the goal of reaching populations exposed to socially vulnerable situations, where health and education conditions may be more compromised. Furthermore, the geographic location facilitated the logistical development of the study, favoring closer ties with the school community.

### Study protocol

During a school meeting, the study objectives were presented to parents, and the contact details of those interested in participating were recorded in a spreadsheet. Subsequently, a message was sent via a messaging app containing a link to a Google Forms form. This form included the Informed Consent Form (ICF) and a question designed to identify the most convenient day and time for the telephone interview.

After confirming participation and signing the ICF, parents were contacted by phone. Up to three contact attempts were made for each registered number. Interviews lasted an average of 25 minutes. At the end of each call, parents were informed that they could be contacted again after 30 days for a follow-up interview (test-retest). This 30-day interval was considered appropriate based on previous studies that also conducted test-retest^(16,17)^.

Data were collected using a sociodemographic and clinical form that aimed to characterize parents and identify the conditions that led to their children’s antibiotic use. The form included variables such as degree of kinship with the child, age, race, marital status, number of children under their care, education level, current occupation, type of housing, family income, government assistance, child’s age at antibiotic use, which antibiotic, and reason/condition that led to its use. To assess parental perception, the Parental Perceptions of Antibiotics Scale (PAPA Scale), duly translated and adapted for use in Brazil, was used^(18)^.

The PAPA Scale was originally developed in English to assess parental perception of the factors that influence their children’s antibiotic use at home. It is a Likert scale with 36 items distributed across six factors: Factor 1 - Knowledge and beliefs (ten items); Factor 2 - Behaviors (six items); Factor 3 - Sources of information (seven items); Factor 4 - Adherence (five items); Factor 5 - Awareness about antibiotics resistance (five items); and Factor 6 - Perceptions of doctors’ prescribing behaviors (three items). Each item is scored from 1 to 5 (1 - Strongly disagree; 2 - Somewhat disagree; 3 - Neither agree nor disagree; 4 - Somewhat agree; 5 - Strongly agree)^(19)^.

For this study, the translated and cross-culturally adapted version for Brazilian Portuguese was used. Scores for each PAPA-Br factor were assessed individually. Lower scores on factors 1, 2, and 4 indicate better parental perception of antibiotic use, as statements in these items are incorrect. Therefore, lower scores reflect better knowledge and beliefs, behaviors, and treatment adherence. For factor 5, higher scores are ideal, indicating better awareness about antibiotic actions, effects, and resistance. Factors 3 and 6 are not scored but are used to understand how parents obtain health-related information and their perceptions of doctors’ prescribing behaviors and the information provided, respectively^(18)^.

### Analysis of results and statistics

Data were organized in a Microsoft Excel for Windows 2010^®^ spreadsheet and analyzed using the Statistical Package for the Social Sciences version 27.0, using descriptive and inferential statistics. PAPA-Br scores were compared according to sociodemographic characteristics. For quantitative variables involving two groups, the nonparametric Mann-Whitney test was used. For comparisons involving three or more groups, the Kruskal-Wallis test was used, followed by Dunn’s post-hoc test, due to the non-normal distribution of the data confirmed by the Kolmogorov-Smirnov test (p<0.001). Finally, Spearman’s correlation coefficient was used to analyze the correlation between scale factors and variables such as parental age, family income, and children’s age. A significance level of 5% was established for all inferential analyses.

## RESULTS

Among participants, young adults (77.0%), female (90.9%), with a mean age of 33.37 + 7.5 years predominated. The majority identified as brown or black (68.4%), reported being married or in a stable relationship (68.4%), having higher education (56.9%), and residing in their own home (77.0%). Income extremes predominated, with 36.8% having an income above three minimum wages (MW) and 24.9% having an income of up to one MW. The majority did not receive government assistance (72.7%).

Children’s mean age was 5.65 + 3.1 years. The main reasons reported for antibiotic use included pharyngitis (28.2%), flu (14.4%), pneumonia (7.7%), sinusitis (7.7%), and fever (6.22%). Other reasons mentioned were cough, otitis, tonsillitis, rhinitis, asthma, and intestinal and urinary infections. Only one child used antibiotics due to a COVID-19 diagnosis.

The most commonly used antibiotics were amoxicillin (30.1%), amoxicillin with potassium clavulanate (28.7%), and azithromycin (19.6%). However, 18.7% of parents did not remember the name of the medication used. The predominant form of administration was oral solution (91.9%).

Table 1 presents parents’ responses to each item of PAPA-Br, considering its six factors. For factors 1 (Knowledge and beliefs), 2 (Behaviors), 4 (Adherence), and 5 (Awareness about antibiotics resistance), most responses were classified as appropriate. However, 14.8% of parents admitted to purchasing antibiotics for their children at pharmacies without a prescription. Regarding sources of information, 28.7% of parents used books and/or scientific literature; 51.2% relied on their own previous experiences; and 37.3% conducted online research. Regarding their perception of doctors’ prescribing behavior, 50.7% of parents reported that professionals did not provide satisfactory information about their children’s health status.

**Table 1:** Distribution of parental responses to items on the Parental Perceptions of Antibiotics Scale, Brazilian version (*Escala de Percepção dos Pais sobre Antibióticos*): Percentages (N=209), Fortaleza, Ceará, Brazil, 2022

Scale items	Totally disagree or partially disagree	Neither agree nor disagree	Partially agree or totally agree
***Fator 1: Conhecimentos e crenças***			
*1 - Antibióticos são necessários para o resfriado comum*	187 (89.4%)	11 (5.3%)	11 (5.3%)
*2 - Antibióticos são necessários para dor na garganta*	93 (44.5%)	27 (12.9%)	89 (42.6%)
*3 - Antibióticos tratam infecções virais*	80 (38.3%)	24 (11.5%)	105 (50.2%)
*4 - Antibióticos podem curar TODOS os tipos de infecções (virais, bacterianas e fúngicas)*	78 (37.3%)	36 (17.2%)	95 (45.5%)
*5 - Quando busco atendimento médico para minha criança com resfriado comum, eu espero uma receita de medicamentos, incluindo antibióticos*	158 (75.6%)	22 (10.5%)	29 (13.9%)
*6 - Antibióticos são úteis no tratamento de resfriados comuns em crianças*	161 (77.1%)	17 (8.1%)	31 (14.8%)
*7 - Crianças com resfriados comuns melhoram mais rápido quando tomam antibióticos*	131 (62.7%)	28 (13.4%)	50 (23.9%)
*8 - Anteriormente, antibióticos curaram os sintomas de resfriado da minha criança*	125 (59.8%)	17 (8.1%)	67 (32.1%)
*9 - Minha criança ficará doente por mais tempo se ela não tomar antibióticos para tosse, resfriado ou sintomas da gripe*	140 (67.0%)	21 (10.0%)	48 (23.0%)
*10 - Se minha criança estiver resfriada ou tossindo é melhor ela tomar um antibiótico para se curar*	155 (74.2%)	21 (10.0%)	33 (15.8%)
***Fator 2: Comportamentos***			
*11 - Antibióticos deveriam ser vendidos sem receita médica*	172 (82.3%)	13 (6.2%)	24 (11.5%)
*12 - Anteriormente, eu parei de dar antibióticos à minha criança porque meus amigos e/ou familiares me aconselharam*	157 (75.1%)	33 (15.8%)	19 (9.1%)
*13 - Eu compro antibióticos para minha criança na farmácia sem receita médica*	173 (82.8%)	5 (2.4%)	31 (14.8%)
*14 - Geralmente, eu guardo antibióticos em casa para usá-los quando forem necessários*	163 (78.0%)	8 (3.8%)	38 (18.2%)
*15 - Anteriormente, eu dei à minha criança um antibiótico sem receita médica quando ela estava com febre alta por alguns dias*	170 (81.3%)	5 (2.4%)	34 (16.3%)
*16 - Anteriormente, eu mudei de médico quando ele não prescreveu antibióticos para minha criança*	196 (93.8%)	5 (2.4%)	8 (3.8%)
***Fator 3: Fontes de informação***			
*17 - Eu obtenho minhas informações relacionadas à saúde com os profissionais da farmácia*	115 (55.0%)	29 (13.9%)	65 (31.1%)
*18 - Eu obtenho minhas informações relacionadas à saúde com enfermeiros(as) e/ou outros profissionais de saúde*	37 (17.7%)	11 (5.3%)	161 (77.0%)
*19 - Eu obtenho minhas informações relacionadas à saúde a partir de livros e/ou literatura científica*	123 (58.9%)	26 (12.4%)	60 (28.7%)
*20 - Eu obtenho minhas informações relacionadas à saúde com familiares e/ou amigos*	121 (57.9%)	21 (10.0%)	67 (32.1%)
*21 - Eu obtenho minhas informações relacionadas à saúde na Internet*	106 (50.7%)	25 (12.0%)	78 (37.3%)
*22 - Eu obtenho minhas informações relacionadas à saúde na mídia: TV, rádio ou jornais*	162 (77.5%)	17 (8.1%)	30 (14.4%)
*23 - Eu obtenho minhas informações relacionadas à saúde da minha própria experiência anterior*	83 (39.7%)	19 (9.1%)	107 (51.2%)
***Fator 4: Adesão***			
*24 - Não é importante seguir rigorosamente doses e horários de antibióticos*	192 (91.9%)	5 (2.4%)	12 (5.7%)
*25 - Deixar de tomar uma ou duas doses de antibióticos não faz muita diferença*	196 (93.8%)	6 (2.9%)	7 (3.3%)
*26 - Se minha criança melhorar, eu posso diminuir a dose dos antibióticos*	189 (90.4%)	7 (3.4%)	13 (6.2%)
*27 - Se a condição de saúde da minha criança for leve, eu dou o antibiótico de acordo com o que acho adequado para a condição dela*	183 (87.6%)	8 (3.8%)	18 (8.6%)
*28 - Anteriormente, eu parei de dar antibiótico à minha criança porque ela se sentia melhor*	170 (81.3%)	12 (5.7%)	27 (13.0%)
***Fator 5: Conscientização sobre resistência aos antibióticos***			
*29 - Antibióticos tratam infecções bacterianas*	17 (8.1%)	20 (9.6%)	172 (82.3%)
*30 - Geralmente, os antibióticos são seguros*	19 (9.1%)	30 (14.4%)	160 (76.5%)
*31 - Antibióticos podem ser prejudiciais à saúde (fazer mal)*	31 (14.8%)	40 (19.1%)	138 (66.1%)
*32 - Alguns microorganismos (bactérias) estão se tornando mais difíceis de tratar com antibióticos*	25 (12.0%)	21 (10.0%)	163 (78.0%)
*33 - Alguns microorganismos (bactérias) podem se tornar resistentes aos antibióticos se forem tomados em doses erradas*	15 (7.2%)	10 (4.8%)	184 (88.0%)
***Fator 6: Percepção dos pais sobre o comportamento de prescrição dos médicos***			
*34 - Eu acho que os médicos receitam muitos antibióticos*	79 (37.8%)	42 (20.1%)	88 (42.1%)
*35 - Médicos não informam bem os pais sobre a condição de saúde de suas crianças*	70 (33.5%)	33 (15.8%)	106 (50.7%)
*36 - Médicos não são bem informados sobre o uso criterioso dos antibióticos*	101 (48.3%)	35 (16.7%)	73 (35.0%)

Table 2 presents a comparison of the scores obtained for each PAPA-Br factor according to parents’ sociodemographic characteristics. Statistically significant differences were identified between the groups analyzed for at least one sociodemographic variable. It should be noted that domains three and six are not represented in the table, as they do not generate numerical scores. These domains aim to investigate, respectively, how parents obtain health-related information and their perceptions of doctors’ prescribing behavior and the information provided.

**Table 2:** Comparative analysis of the median of the Parental Perceptions of Antibiotics Scale, Brazilian version (*Escala de Percepção dos Pais sobre Antibióticos*) factors and parents’ sociodemographic characteristics (N=209), Fortaleza, Ceará, Brazil, 2022

Variables	Factor 1	Factor 2	Factor 4	Factor 5
	Median	Median	Median	Median
Kinship				
Mother	20.50	8.00	5.00	22.00
Father	22.00	9.00	5.00	20.00
*p* value	0.296^^*^^	0.107^^*^^	0.909^^*^^	0.165^^*^^
Age group (years)				
19-29	22.00	9.00	5.00	20.00
30-39	20.00	8.00	5.00	22.00
40-52	21.00	8.50	5.00	23.00
*p* value	0.362^†^	0.249^†^	0.946^†^	0.015^†^
Ethnicity				
White	18.00	7.00	5.00	23.00
Brown/black	22.00	9.00	5.00	21.00
*p* value	0.013^^*^^	0.163^^*^^	0.265^^*^^	0.155^^*^^
Marital status				
With partner	20.00	8.00	5.00	22.00
Without partner	23.50	8.50	5.00	21.00
*p* value	0.011^^*^^	0.176^^*^^	0.458^^*^^	0.335^^*^^
Education				
Elementary school	29.50	12.50	5.50	20.50
High school	22.00	10.00	5.00	21.00
Higher education	20.00	7.00	5.00	22.00
*p* value	0.001^†^	0.004^†^	0.017^†^	0.093^†^
Occupation				
Healthcare professional	16.00	6.00	5.00	24.00
Education professional	18.00	8.00	5.00	22.00
Self-employed	23.00	10.00	5.00	21.00
Housewife	22.00	9.00	5.00	21.00
Other	21.00	8.00	5.00	21.00
*p* value	0.001^†^	0.076^†^	0.067^†^	0.036^†^
Income (MW)				
Up to 1	24.00	10.00	5.00	20.00
From 1.1 - 2	22.00	9.50	5.00	21.00
From 2.1 - 3	19.50	9.50	5.00	21.00
> 3	18.00	6.00	5.00	23.00
*p* value	<0.001^†^	0.002^†^	0.011^†^	<0.001^†^

factor 1 - Knowledge and beliefs; factor 2 - Behaviors; factor 4 - Adherence; factor 5 - Awareness about antibiotics resistance; MW - minimum wage in force in 2022 (R$ 1,212.00);

* Mann-Whitney test; †Kruskal-Wallis test.

In factor 1, Parental knowledge and beliefs about antibiotics, it was found, based on univariate analysis, that parents who self-declared as brown/black and who did not have a partner achieved higher scores on PAPA-Br, demonstrating that they have less knowledge and beliefs about the use of antibiotics in pediatrics.

Education, occupation, and income were variables in which a statistical difference was identified in the scores achieved between the groups in factor 1 of PAPA-Br. After performing Dunn’s post-hoc test, it was observed that: parents with higher education presented better knowledge and belief, when compared to those with elementary education (p=0.001); parents who were healthcare professionals presented better knowledge than those who had another occupation (p=0.033) or who were housewives (p=0.013) or self-employed (p=0.001); parents with a family income above three MW presented better knowledge and belief than parents with an income of up to one MW (p=<0.001) or that varied between one and two MW (p=0.032).

Factor 2, Behaviors, showed statistically significant differences between groups in the education and income variables. After post-hoc analysis, it was found that parents with higher education presented better results regarding antibiotic use behavior, when compared to those with secondary education (p=0.016). In the analysis of association with income, it was found that parents with a family income above three MW presented more satisfactory scores compared to those with an income of up to one MW (p=0.002) or income between one and two MW (p=0.048).

In factor 4, Treatment adherence, as in factor 2, a difference in medians in PAPA-Br scores was observed between groups for the education and income variables. However, after performing Dunn’s post-hoc test, with adjusted p-value, a statistical difference was observed only for the income variable, with parents with an income above three MW showing better treatment adherence when compared to those with an income of up to one MW (p=0.007).

Factor 5, Parental awareness about antibiotics resistance, showed significant differences in medians between groups for the age, occupation, and income variables. After performing Dunn’s post-hoc test, it was found that: parents aged 30 to 39 years were more aware of antibiotics resistance when compared to those aged 19 to 29 years (p=0.015); parents who were healthcare professionals obtained better scores on this factor when compared to those in other roles (except education professional, homemaker, and self-employed) (p=0.023).

A correlation analysis between parents’ age, income and children’s age variables was also performed, as shown in Table 3.

**Table 3:** Spearman correlation analysis of sociodemographic characteristics of parents and children with factors from the Parental Perceptions of Antibiotics Scale, Brazilian version (*Escala de Percepção dos Pais sobre Antibióticos*) (N=209), Fortaleza, Ceará, Brazil, 2022

Factor	Parents’ age		Income		Children’s age	
	*r_s_*	p	r_s_	p	r_s_	p
Factor 1	- 0.084	0.226	-0.328	<0.001	0.086	0.216
Factor 2	- 0.035	0.617	-0.266	<0.001	0.078	0.261
Factor 4	0.005	0.938	-0.261	<0.001	-0.049	0.478
Factor 5	0.202	0.003	0.292	<0.001	0.012	0.868

*factor 1 - Knowledge and beliefs; factor 2 - Behaviors; factor 4 - Adherence; factor 5 - Awareness about antibiotics resistance; rs - Spearman’s correlation coefficient; p - test significance value.*

The correlation between parental age and factor 5 was statistically significant (n=209; rs=0.202; p=0.003), suggesting that awareness about antibiotics resistance tends to increase with parental age. The income variable demonstrated statistically significant correlations with the following factors: factor 1 (n=209; r_s_ = -0.328; p < 0.001), indicating that as income decreases, knowledge about antibiotics decreases; factor 2 (n=209; r_s_ = -0.266; p < 0.001), suggesting that as income decreases, antibiotic-related behaviors may become less favorable; factor 4 (n=209; r_s_ = -0.261; p < 0.001), suggesting that as income decreases, adherence to antibiotic treatment may decrease; and factor 5 (n=209; r_s_ = 0.292; p < 0.001), indicating that as income increases, awareness about antibiotics resistance tends to increase. However, it is important to note that, although statistically significant, the strength of the correlations was weak.

## DISCUSSION

Parental perception of antibiotic use in their children has been extensively investigated due to the significant impact of the indiscriminate use of these medications on children’s health. The assessment conducted using the PAPA-Br questionnaire, combined with sociodemographic and clinical data analysis, demonstrated consistency with the findings of international studies.

The predominance of mothers among the participants is consistent with other studies that investigated parents’ knowledge and perceptions about antibiotic use in children, in which mothers constituted the majority of the sample^(19-21)^. This result may be related to cultural norms that often assign women the primary role in childcare^(22)^. Likewise, children’s mean age is similar to data from a study carried out in rural communities in Peru^(23)^, reinforcing the importance of investigations that consider age variables in the use of antibiotics.

In this study, medication use was primarily due to respiratory infections. Similar results were found in a study conducted in Australia, in which parents reported that antibiotics are indicated for colds and flu. It was also found that parents with this belief were more likely to have administered antibiotics to their children in the 12 months prior to the study^(24)^. During the translation and cross-cultural adaptation stage of the PAPA Scale into Brazilian Portuguese, parents also reported that sore throat, flu, pneumonia, and bronchitis were the main causes of antibiotic use by their children^(18)^.

There is a common misconception that antibiotics are effective in treating any type of infection, including viral infections. This misconception can lead to inappropriate antibiotic use, contributing to bacterial resistance, a serious public health problem. Studies point to a direct link between bacterial resistance and factors such as inappropriate prescribing and incorrect use of antibiotics^(25,26)^.

This trend was also found in Turkey, where a significant proportion of parents believed that antibiotics were effective in treating both bacterial and viral infections^(27)^. Such misperceptions exacerbate inappropriate use, especially when combined with the still-common practice of self-medication. This situation has been frequently observed in countries with less control over the marketing of these medications, and can have negative consequences for developing children, such as increased susceptibility to infections^(9,28)^.

In contrast, a study conducted in the United Kingdom found that mothers sought a medical prescription before administering antibiotics to their children^(29)^. In Sweden, the purchase of antibiotics without a prescription is low, reflecting greater public awareness about bacterial resistance and stricter controls on the sale of these drugs^(30)^. This difference is presumably attributed to more effective public policies in developed countries.

The sources of information most cited by parents about antibiotic use are healthcare professionals and their own previous experiences. A qualitative study in Zomba, Malawi, with low-income mothers revealed that healthcare professionals are the main sources of information about antibiotics, although some mothers also mentioned friends, relatives, schools, and the media^(31)^. This pattern was observed in a study in Lebanon, where most participants reported acquiring health knowledge through previous experiences^(20)^.

Despite the growing use of the internet as a source of information, it is observed that the content accessed often lacks technical basis, sometimes being disseminated by social media and influencers without training in the health field. This scenario contributes to misinformation and reinforces the need for appropriate actions, especially in community and school settings^(32-34)^.

Education has been shown to be a factor strongly associated with parental perception and behavior regarding antibiotic use. Studies indicate that the likelihood of antibiotic use is higher among mothers with less education, while more appropriate practices are observed among parents with higher education and health literacy^(20,35)^. Furthermore, parents with limited understanding of how antibiotics work often have poor adherence to prescribed treatment and improper disposal of medications, even after medical advice^(36)^.

In Bangladesh, variables such as age, gender, educational level, employment status, and household income were found to be associated with inappropriate antibiotic use. These findings highlight the importance of educational initiatives focused on responsible antibiotic use and reducing antimicrobial resistance^(37,38)^. Similarly, parents with lower educational levels may have limited understanding of the potential harms and long-term effects associated with inappropriate antibiotic use^(20,35,39)^.

A concerning behavior reported by some participants was the storage of leftover antibiotics for future use, suggesting misconceptions about reusing prescription medications. In Shanghai, a study revealed that self-medication with stored antibiotics is common among parents^(40)^.

These findings highlight that healthcare professionals play a crucial role in educating parents about the importance of using these medications appropriately. Well-structured and targeted educational interventions, combined with television and social media campaigns, can help reduce the tendency to self-medicate and reduce parental pressure on healthcare professionals to prescribe antibiotics when their children are sick^(21)^.

In this context, nurses play a strategic role in promoting the rational use of medications at different levels of healthcare. In primary care, they act as educational agents, using accessible language and teaching resources in individual and collective initiatives. When aligned with the sociocultural and economic context of communities, interventions such as discussion groups, role-plays, videos, and games become more effective and accessible.

In both hospital and home settings, nurses contribute to the safe continuity of care by guiding parents and guardians at discharge and during home monitoring. By raising awareness about the risks of self-medication and premature discontinuation of treatment, nurses strengthen therapeutic adherence and prevent adverse events^(41)^. Thus, the importance of educational actions conducted by nurses integrated into the different phases of care and adapted to the realities of patients and their families is reinforced.

### Study limitations

This study has some limitations, such as restricting data collection to the capital of Ceará, which prevents comparison with populations from other regions of Brazil. Furthermore, the snowball recruitment method used may have introduced selection bias, limiting the diversity of the sample. This approach may lead to a sample that is less representative of the general population, which could impact the findings. Future studies should consider including samples from different regional contexts to assess the consistency of results.

### Contributions to health and nursing

The strengths of this study include being the first to use PAPA-Br, a version culturally adapted to Brazil, which allowed for an analysis of Brazilian parental perception of antibiotic use. By considering specific cultural factors that may influence the use of these medications, this work offers a novel and relevant contribution to the field. Furthermore, collecting data in a capital city in northeastern Brazil broadens the study’s impact, providing valuable insights into a region often underrepresented in scientific research, strengthening the foundation for the development of future educational interventions tailored to local needs.

Identifying the factors that influence parents’ inappropriate or incorrect antibiotic use can help develop specific educational strategies that improve parents’ knowledge, beliefs, behaviors, adherence, and awareness. These strategies can also facilitate effective communication between healthcare professionals and parents, contributing to more effective therapy and safe antibiotic administration, with positive impacts on both child health and the reduction of antimicrobial resistance.

In the context of nursing practice, the study results provide support for nurses in developing and implementing educational strategies targeted to the population’s needs, especially in primary care settings. Professionals can use the findings to structure educational initiatives for caregivers, focusing on promoting the rational use of antibiotics, clarifying the difference between viral and bacterial infections, and raising awareness about the risks of misuse of these medications. Furthermore, nurses can work with multidisciplinary teams to implement safe prescribing protocols and monitor treatment adherence. Thus, the study contributes to strengthening evidence-based practice and the role of nursing in preventing antimicrobial resistance.

## CONCLUSIONS

The results showed that parents’ decisions about their children’s antibiotic use at home are influenced by their beliefs, practices based on previous experiences, and educational level, highlighting the need for targeted interventions. The research contributes to health and society by reinforcing the importance of educational strategies that promote knowledge and appropriate behaviors, in addition to strengthening communication between parents and healthcare professionals. These measures are essential to ensure the rational use of antibiotics, minimizing health risks for children and combating antimicrobial resistance.

## Data Availability

The research data are available within the article.
