# Supplementary material for: Parental perception of antibiotic use in children at home: a cross-sectional study
Source: Rev Bras Enferm. 2025 Nov 17;78(Suppl 3):e20250030. doi: 10.1590/0034-7167-2025-0030 (PMC12622974; doi:10.1590/0034-7167-2025-0030)
Supplement: Translation

# Translation: Percepção dos pais sobre o uso de antibióticos em crianças no domicílio: estudo transversal

**DOI:** 10.1590/0034-7167-2025-0030pt

**Authors:** Glaubervania Alves Lima, Francisca Elisângela Teixeira Lima, Luis Angel Cendejas Medina, Sara Emilly Lima Sombra, Fernanda Jorge Magalhães, Sabrina de Souza Gurgel Florencio, Maria Gabriela Miranda Fontenele, Lorena Pinheiro Barbosa

**Affiliations:** I Universidade Federal do Ceará. Fortaleza, Ceará, Brasil; II Universidade de São Paulo. Ribeirão Preto, São Paulo, Brasil; III Universidade Estadual do Ceará. Fortaleza, Ceará, Brasil

**Keywords:** Saúde da Criança, Infecções Respiratórias, Antibacterianos, Enfermagem, Percepção.

## Abstract

**Objetivos:**

avaliar a percepção dos pais acerca dos fatores que influenciam o uso de antibióticos por seus filhos no domicílio e verificar a associação com características sociodemográficas.

**Métodos:**

estudo descritivo, transversal, realizado com 209 pais de crianças menores de 12 anos que utilizaram antibióticos em domicílio. Aplicaram-se um instrumento de caracterização sociodemográfica e clínica e a Escala de Percepção dos Pais sobre Antibióticos. Realizaram-se os testes de Mann-Whitney, Kruskal-Wallis e Spearman para análise dos dados.

**Resultados:**

a maioria dos participantes eram mães (90,9%). Pais com ensino fundamental e renda inferior a um salário mínimo apresentaram maiores medianas nos fatores da escala 1- Conhecimentos e crenças, 2- Comportamentos, e 3- Adesão, indicando menor compreensão sobre o uso correto de antibióticos.

**Conclusões:**

a percepção dos pais sobre os fatores que influenciam a administração de antibióticos às crianças em domicílio está diretamente associada ao uso correto da medicação e aos fatores sociodemográficos.

## INTRODUÇÃO

As infecções respiratórias e digestivas, tais como gripes, resfriados, pneumonias, otites e gastroenterites, são comuns na infância^(1,2)^. A entrada das crianças no ambiente escolar aumenta sua suscetibilidade a essas infecções, tornando-as mais expostas ao uso de medicamentos, incluindo antibióticos^(3,4)^.

Embora os antibióticos sejam essenciais para tratar infecções bacterianas, seu uso inadequado ou excessivo pode acarretar graves consequências. Entre os problemas associados estão o desenvolvimento de resistência bacteriana, o aumento das doenças crônicas e o impacto nos custos dos serviços de saúde^(5-7)^. Esses efeitos são ainda mais preocupantes em crianças, influenciando negativamente seu crescimento e saúde a longo prazo^(2)^. Por isso, o uso desnecessário e inadequado de antibióticos é considerado um dos maiores desafios de saúde pública global^(8,9)^.

Diversos fatores contribuem para o uso indevido de antibióticos, incluindo prescrições médicas inadequadas, falta de conhecimento e atitudes equivocadas dos pais ou responsáveis^(10)^. A automedicação e a compra de medicamentos sem prescrição médica persistem como práticas comuns, especialmente em contextos onde o acesso aos serviços de saúde é limitado^(11)^. Além disso, a percepção negativa das pessoas em relação à interação médico-paciente, a insatisfação com o atendimento e experiências prévias bem-sucedidas com o uso de antibióticos podem reforçar condutas inadequadas^(12)^. Contudo, esse fenômeno também está fortemente relacionado a problemas estruturais de saúde pública, como a dificuldade de acesso aos serviços médicos e escassez de profissionais, especialmente em áreas mais vulneráveis. Ademais, fatores socioeconômicos, como baixa escolaridade, renda insuficiente e acesso limitado a informações confiáveis sobre o uso racional de medicamentos, favorecem práticas inadequadas no manejo de doenças infantis^(4,10)^.

Portanto, diante dessas constatações e da lacuna existente na literatura brasileira sobre o entendimento dos cuidadores quanto aos riscos associados à administração de antibióticos a seus filhos, surgiu a inquietação que fundamentou o presente estudo. Diante desse cenário, compreender como os pais percebem o uso de antibióticos por seus filhos é fundamental para identificar os fatores que influenciam práticas inadequadas e embasar o desenvolvimento de estratégias educativas voltadas ao manejo domiciliar da antibioticoterapia. Tais intervenções podem promover uma comunicação mais eficaz entre cuidadores e profissionais de saúde, estabelecer regimes terapêuticos adequados e prevenir falhas no tratamento que comprometam a saúde das crianças^(13,14)^.

Dessa forma, este estudo contribui para a prática clínica e acadêmica ao fornecer subsídios para o desenvolvimento de tecnologias e estratégias educativas mais alinhadas às necessidades e realidades das famílias. Ao adotar a perspectiva dos cuidadores, a pesquisa aprofunda a análise dos fatores que motivam práticas inadequadas no uso domiciliar de antibióticos, colaborando para a promoção de políticas e ações que favoreçam o uso racional desses medicamentos e a segurança no cuidado infantil.

## OBJETIVOS

Avaliar a percepção dos pais acerca dos fatores que influenciam o uso de antibióticos por seus filhos no domicílio e verificar a associação com características sociodemográficas.

## MÉTODOS

### Aspectos éticos

O estudo foi submetido e aprovado pelo Comitê de Ética em Pesquisa, em conformidade com os preceitos éticos da Resolução n^o^ 466/2012 do Conselho Nacional de Saúde.

### Tipo de estudo

Estudo transversal, descritivo, realizado na cidade de Fortaleza, Ceará, no período de setembro a novembro de 2022. A elaboração do manuscrito seguiu as diretrizes do *STrengthening the Reporting of OBservational studies in Epidemiology* ^(15)^.

### População, amostra e critérios de seleção

Para o cálculo do tamanho amostral, estimou-se uma proporção (P) de 50% de pais de crianças que já fizeram uso de antibióticos em domicílio, com nível de confiança de 95% (z = 1,96) e erro amostral absoluto de 5%. Esses parâmetros foram aplicados à fórmula para populações finitas, considerando um N de 450 pais cadastrados nas duas escolas participantes, resultando em um tamanho amostral (n) de 207 participantes:


$$n=\frac{N\times Z^{2}\times p(1-p)}{e^{2}(N-1)+Z^{2}\times p(1-p)}$$

No entanto, durante a coleta de dados, 209 pais responderam ao instrumento, compondo a amostra final do estudo, que atendeu aos seguintes critérios de inclusão: ser pai ou mãe de criança com menos de 12 anos de idade que tenha feito uso de antibióticos em domicílio nos seis meses anteriores à coleta de dados e possuir um *smartphone* com acesso à internet. Foram excluídos indivíduos com déficits cognitivos ou que não atenderam às três tentativas de contato.

A coleta de dados foi realizada em duas escolas de educação infantil e ensino fundamental da cidade de Fortaleza, Ceará. Para ampliar o alcance da amostra e incluir pais cujos filhos não estavam matriculados nessas escolas, mas que haviam utilizado antibióticos em domicílio, adotou-se a estratégia de amostragem por bola de neve, na qual os participantes indicaram outros pais com perfil compatível para participar do estudo.

Administrativamente, Fortaleza está dividida em 12 Secretarias Executivas Regionais (SER). As escolas selecionadas estão situadas em um bairro pertencente à SER IX, considerada uma das áreas mais vulneráveis da cidade. Essa área apresenta desafios significativos do ponto de vista socioeconômico, como baixos Índices de Desenvolvimento Humano e alta densidade populacional. A escolha dessas instituições de ensino se deu de forma intencional, justificando-se pela intenção de alcançar populações expostas a situações de vulnerabilidade social, nas quais as condições de saúde e educação podem estar mais comprometidas. Além disso, a localização geográfica facilitou o desenvolvimento logístico do estudo, favorecendo a aproximação com a comunidade escolar.

### Protocolo do estudo

Durante uma reunião escolar, os objetivos do estudo foram apresentados aos pais, e os contatos daqueles interessados em participar foram registrados em uma planilha. Posteriormente, foi enviada uma mensagem por meio de um aplicativo de mensagens contendo o *link* para um formulário no *Google Forms*. Este formulário incluía o Termo de Consentimento Livre e Esclarecido (TCLE) e uma pergunta destinada a identificar o dia e o horário mais convenientes para a realização da entrevista telefônica.

Após a confirmação da participação e assinatura do TCLE, os pais foram contatados por meio de chamadas telefônicas. Foram realizadas até três tentativas de contato para cada número registrado. As entrevistas tiveram duração média de 25 minutos. Ao final de cada ligação, os pais foram informados de que poderiam ser contatados novamente após 30 dias para uma entrevista de acompanhamento (teste-reteste). Esse intervalo de 30 dias foi considerado apropriado com base em estudos anteriores que também realizaram a aplicação do teste-reteste^(16,17)^.

Os dados foram coletados por meio de um formulário sociodemográfico e clínico que visava caracterizar os pais e identificar as condições que levaram ao uso de antibióticos pela criança. O formulário incluía variáveis como grau de parentesco com a criança, idade, cor, estado civil, número de filhos em sua responsabilidade, escolaridade, ocupação atual, tipo de moradia, renda familiar, auxilio do governo, idade da criança ao fazer uso do antibiótico, qual antibiótico e o motivo/doença que levou ao seu uso. Para avaliação da percepção dos pais, utilizou-se a Escala de Percepção Parental sobre Antibióticos (PAPA *Scale*) devidamente traduzida e adaptada para uso no Brasil^(18)^.

A PAPA *Scale* foi originalmente desenvolvida em inglês com o objetivo de avaliar a percepção dos pais sobre os fatores que influenciam o uso de antibióticos pelos filhos em domicílio. Trata-se de escala Likert com 36 itens distribuídos em seis fatores: fator 1 - Conhecimento e crenças (dez itens); fator 2 - Comportamentos (seis itens); fator 3 - Fontes de informação (sete itens); fator 4 - Adesão (cinco itens); fator 5 - Conscientização sobre a resistência aos antibióticos (cinco itens); e fator 6 - Percepção dos pais sobre o comportamento de prescrição dos médicos (três itens). Cada item é pontuado de 1 a 5 (1 - Discordo totalmente; 2 - Discordo parcialmente; 3 - Nem concordo nem discordo; 4 - Concordo parcialmente; 5 - Concordo totalmente)^(19)^.

Para este estudo, utilizou-se a versão traduzida e adaptada transculturalmente para o português brasileiro. Os escores de cada fator da PAPA-Br foram avaliados individualmente. Escores mais baixos nos fatores 1, 2 e 4 indicam melhor percepção dos pais sobre o uso de antibióticos, pois as afirmações nesses itens estão incorretas. Portanto, escores mais baixos refletem melhor conhecimento e crenças, comportamentos, e adesão ao tratamento. Para o fator 5, escores mais altos são ideais, indicando melhor conscientização sobre as ações, efeitos e resistência aos antibióticos. Os fatores 3 e 6 não são pontuados, mas são usados para entender como os pais obtêm informações relacionadas à saúde e suas percepções sobre os comportamentos de prescrição dos médicos e as informações fornecidas, respectivamente^(18)^.

### Análise dos resultados e estatística

Os dados foram organizados em planilha eletrônica do *Microsoft Excel for Windows 2010* ^®^ e analisados no *Statistical Package for the Social Sciences*, versão 27.0, por meio de estatística descritiva e inferencial. Os escores obtidos no PAPA-Br foram comparados segundo as características sociodemográficas. Para variáveis quantitativas envolvendo dois grupos, foi utilizado o teste não paramétrico de Mann-Whitney. Para comparações envolvendo três ou mais grupos, foi utilizado o teste de Kruskal-Wallis, seguido do teste *post-hoc* de Dunn, devido à distribuição não normal dos dados confirmada pelo teste de Kolmogorov-Smirnov (p<0,001). Por fim, foi utilizado o coeficiente de correlação de Spearman para analisar a correlação entre os fatores da escala e variáveis como idade dos pais, renda familiar e idade dos filhos. Foi estabelecido um nível de significância de 5% para todas as análises inferenciais.

## RESULTADOS

Entre os participantes, predominaram adultos jovens (77,0%), do sexo feminino (90,9%), com média de idade de 33,37 + 7,5 anos. A maioria identificou-se como pardo ou preto (68,4%), relatou estar casado ou em união estável (68,4%), possuir ensino superior (56,9%) e residir em casa própria (77,0%). Predominaram os extremos de renda, sendo 36,8% com renda superior a três salários mínimos (SM) e 24,9% com renda de até um SM. A maioria não recebia auxílio do governo (72,7%).

A média de idade das crianças foi de 5,65 + 3,1 anos. Os principais motivos relatados para o uso de antibióticos incluíram faringite (28,2%), gripe (14,4%), pneumonia (7,7%), sinusite (7,7%) e febre (6,22%). Outros motivos mencionados foram tosse, otite, amidalite, rinite, asma e infecções intestinais e urinárias. Apenas uma criança utilizou antibióticos em razão de diagnóstico de COVID-19.

Os antibióticos mais utilizados foram amoxicilina (30,1%), amoxicilina com clavulanato de potássio (28,7%) e azitromicina (19,6%). Contudo, 18,7% dos pais não se recordaram do nome do medicamento utilizado. A forma de administração predominante foi a solução oral (91,9%).

Na Tabela 1, são apresentadas as respostas dos pais para cada item da PAPA-Br, considerando seus seis fatores. Para os fatores 1 (Conhecimento e crenças), 2 (Comportamentos), 4 (Adesão) e 5 (Consciência da resistência aos antibióticos), a maioria das respostas foi classificada como apropriada. No entanto, 14,8% dos pais admitiram comprar antibióticos para os filhos em farmácias sem receita. Quanto às fontes de informação, 28,7% dos pais recorreram a livros e/ou literatura científica; 51,2% confiaram em suas próprias experiências anteriores; e 37,3% realizaram pesquisas na internet. Em relação à percepção sobre o comportamento de prescrição dos médicos, 50,7% dos pais relataram que os profissionais não forneceram informações satisfatórias sobre a condição de saúde de seus filhos.

**Tabela 1:** Distribuição das respostas dos pais aos itens da Escala de Percepção dos Pais sobre Antibióticos, versão em português: Porcentagens (N=209), Fortaleza, Ceará, Brasil, 2022

Itens da escala	Discordo totalmente ou discordo parcialmente	Nem concordo e nem discordo	Concordo parcialmente ou concordo totalmente
**Fator 1: Conhecimentos e crenças**			
1 - Antibióticos são necessários para o resfriado comum	187 (89,4%)	11 (5,3%)	11 (5,3%)
2 - Antibióticos são necessários para dor na garganta	93 (44,5%)	27 (12,9%)	89 (42,6%)
3 - Antibióticos tratam infecções virais	80 (38,3%)	24 (11,5%)	105 (50,2%)
4 - Antibióticos podem curar TODOS os tipos de infecções (virais, bacterianas e fúngicas)	78 (37,3%)	36 (17,2%)	95 (45,5%)
5 - Quando busco atendimento médico para minha criança com resfriado comum, eu espero uma receita de medicamentos, incluindo antibióticos	158 (75,6%)	22 (10,5%)	29 (13,9%)
6 - Antibióticos são úteis no tratamento de resfriados comuns em crianças	161 (77,1%)	17 (8,1%)	31 (14,8%)
7 - Crianças com resfriados comuns melhoram mais rápido quando tomam antibióticos	131 (62,7%)	28 (13,4%)	50 (23,9%)
8 - Anteriormente, antibióticos curaram os sintomas de resfriado da minha criança	125 (59,8%)	17 (8,1%)	67 (32,1%)
9 - Minha criança ficará doente por mais tempo se ela não tomar antibióticos para tosse, resfriado ou sintomas da gripe	140 (67,0%)	21 (10,0%)	48 (23,0%)
10 - Se minha criança estiver resfriada ou tossindo é melhor ela tomar um antibiótico para se curar	155 (74,2%)	21 (10,0%)	33 (15,8%)
**Fator 2: Comportamentos**			
11 - Antibióticos deveriam ser vendidos sem receita médica	172 (82,3%)	13 (6,2%)	24 (11,5%)
12 - Anteriormente, eu parei de dar antibióticos à minha criança porque meus amigos e/ou familiares me aconselharam	157 (75,1%)	33 (15,8%)	19 (9,1%)
13 - Eu compro antibióticos para minha criança na farmácia sem receita médica	173 (82,8%)	5 (2,4%)	31 (14,8%)
14 - Geralmente, eu guardo antibióticos em casa para usá-los quando forem necessários	163 (78,0%)	8 (3,8%)	38 (18,2%)
15 - Anteriormente, eu dei à minha criança um antibiótico sem receita médica quando ela estava com febre alta por alguns dias	170 (81,3%)	5 (2,4%)	34 (16,3%)
16 - Anteriormente, eu mudei de médico quando ele não prescreveu antibióticos para minha criança	196 (93,8%)	5 (2,4%)	8 (3,8%)
**Fator 3: Fontes de informação**			
17 - Eu obtenho minhas informações relacionadas à saúde com os profissionais da farmácia	115 (55,0%)	29 (13,9%)	65 (31,1%)
18 - Eu obtenho minhas informações relacionadas à saúde com enfermeiros(as) e/ou outros profissionais de saúde	37 (17,7%)	11 (5,3%)	161 (77,0%)
19 - Eu obtenho minhas informações relacionadas à saúde a partir de livros e/ou literatura científica	123 (58,9%)	26 (12,4%)	60 (28,7%)
20 - Eu obtenho minhas informações relacionadas à saúde com familiares e/ou amigos	121 (57,9%)	21 (10,0%)	67 (32,1%)
21 - Eu obtenho minhas informações relacionadas à saúde na Internet	106 (50,7%)	25 (12,0%)	78 (37,3%)
22 - Eu obtenho minhas informações relacionadas à saúde na mídia: TV, rádio ou jornais	162 (77,5%)	17 (8,1%)	30 (14,4%)
23 - Eu obtenho minhas informações relacionadas à saúde da minha própria experiência anterior	83 (39,7%)	19 (9,1%)	107 (51,2%)
**Fator 4: Adesão**			
24 - Não é importante seguir rigorosamente doses e horários de antibióticos	192 (91,9%)	5 (2,4%)	12 (5,7%)
25 - Deixar de tomar uma ou duas doses de antibióticos não faz muita diferença	196 (93,8%)	6 (2,9%)	7 (3,3%)
26 - Se minha criança melhorar, eu posso diminuir a dose dos antibióticos	189 (90,4%)	7 (3,4%)	13 (6,2%)
27 - Se a condição de saúde da minha criança for leve, eu dou o antibiótico de acordo com o que acho adequado para a condição dela	183 (87,6%)	8 (3,8%)	18 (8,6%)
28 - Anteriormente, eu parei de dar antibiótico à minha criança porque ela se sentia melhor	170 (81,3%)	12 (5,7%)	27 (13,0%)
**Fator 5: Conscientização sobre resistência aos antibióticos**			
29 - Antibióticos tratam infecções bacterianas	17 (8,1%)	20 (9,6%)	172 (82,3%)
30 - Geralmente, os antibióticos são seguros	19 (9,1%)	30 (14,4%)	160 (76,5%)
31 - Antibióticos podem ser prejudiciais à saúde (fazer mal)	31 (14,8%)	40 (19,1%)	138 (66,1%)
32 - Alguns microorganismos (bactérias) estão se tornando mais difíceis de tratar com antibióticos	25 (12,0%)	21 (10,0%)	163 (78,0%)
33 - Alguns microorganismos (bactérias) podem se tornar resistentes aos antibióticos se forem tomados em doses erradas	15 (7,2%)	10 (4,8%)	184 (88,0%)
**Fator 6: Percepção dos pais sobre o comportamento de prescrição dos médicos**			
34 - Eu acho que os médicos receitam muitos antibióticos	79 (37,8%)	42 (20,1%)	88 (42,1%)
35 - Médicos não informam bem os pais sobre a condição de saúde de suas crianças	70 (33,5%)	33 (15,8%)	106 (50,7%)
36 - Médicos não são bem informados sobre o uso criterioso dos antibióticos	101 (48,3%)	35 (16,7%)	73 (35,0%)

A Tabela 2 apresenta a comparação dos escores obtidos para cada fator da PAPA-Br de acordo com as características sociodemográficas dos pais. Foram identificadas diferenças estatisticamente significativas entre os grupos analisados para pelo menos uma variável sociodemográfica. Ressalta-se que os domínios três e seis não estão representados na tabela, uma vez que não geram escores numéricos. Esses domínios têm como objetivo investigar, respectivamente, como os pais obtêm informações relacionadas à saúde e suas percepções sobre o comportamento de prescrição dos médicos e as informações fornecidas.

**Tabela 2:** Análise comparativa da mediana dos fatores da Escala de Percepção dos Pais sobre Antibióticos, versão em português, e as características sociodemográficas dos pais (N=209), Fortaleza, Ceará, Brasil, 2022

Variáveis	Fator 1	Fator 2	Fator 4	Fator 5
	Mediana	Mediana	Mediana	Mediana
Parentesco				
Mãe	20,50	8,00	5,00	22,00
Pai	22,00	9,00	5,00	20,00
Valor de *p*	0,296^^*^^	0,107^^*^^	0,909^^*^^	0,165^^*^^
Faixa etária (anos)				
19-29	22,00	9,00	5,00	20,00
30-39	20,00	8,00	5,00	22,00
40-52	21,00	8,50	5,00	23,00
Valor de *p*	0,362^†^	0,249^†^	0,946^†^	0,015^†^
Etnia				
Branca	18,00	7,00	5,00	23,00
Parda/preta	22,00	9,00	5,00	21,00
Valor de *p*	0,013^^*^^	0,163^^*^^	0,265^^*^^	0,155^^*^^
Estado civil				
Com parceiro	20,00	8,00	5,00	22,00
Sem parceiro	23,50	8,50	5,00	21,00
Valor de *p*	0,011^^*^^	0,176^^*^^	0,458^^*^^	0,335^^*^^
Escolaridade				
Ensino fundamental	29,50	12,50	5,50	20,50
Ensino médio	22,00	10,00	5,00	21,00
Ensino superior	20,00	7,00	5,00	22,00
Valor de *p*	0,001^†^	0,004^†^	0,017^†^	0,093^†^
Ocupação				
Profissional de saúde	16,00	6,00	5,00	24,00
Profissional de educação	18,00	8,00	5,00	22,00
Autônomo	23,00	10,00	5,00	21,00
Dona de casa	22,00	9,00	5,00	21,00
Outros	21,00	8,00	5,00	21,00
Valor de *p*	0,001^†^	0,076^†^	0,067^†^	0,036^†^
Renda (SM)				
Até 1	24,00	10,00	5,00	20,00
De 1,1 - 2	22,00	9,50	5,00	21,00
De 2,1 - 3	19,50	9,50	5,00	21,00
> 3	18,00	6,00	5,00	23,00
Valor de *p*	<0,001^†^	0,002^†^	0,011^†^	<0,001^†^

fator 1 - Conhecimento e crenças; fator 2 - Comportamentos; fator 4 - Adesão; fator 5 - Conscientização sobre resistência aos antibióticos; SM - salário mínimo vigente no ano de 2022 (R$ 1.212,00);

* teste de Mann-Whitney; †teste de Kruskal-Wallis.

No fator 1, Conhecimentos e crenças dos pais acerca dos antibióticos, verificou-se, a partir da análise univariada, que pais autodeclarados pardos/pretos e que não possuíam companheiro alcançaram maiores escores na PAPA-Br, demonstrando possuir menor conhecimento e crenças sobre o uso de antibióticos na pediatria.

A escolaridade, ocupação e renda foram variáveis em que foi identificada diferença estatística nos escores alcançados entre os grupos no fator 1 da PAPA-Br. Após a realização do *post-hoc* de Dunn, observou-se que: pais com nível superior apresentaram melhor conhecimento e crença, quando comparados àqueles com nível fundamental (p=0,001); pais que eram profissionais de saúde apresentaram melhor conhecimento em detrimento dos que possuíam outra ocupação (p=0,033) ou que eram donas de casa (p=0,013) ou autônomos (p=0,001); pais com renda familiar superior a três SM apresentaram melhor conhecimento e crença que os pais com renda de até um SM (p=<0,001) ou que variasse entre um e dois SM (p=0,032).

O fator 2, Comportamentos, apresentou diferenças significativas estatisticamente entre grupos nas variáveis escolaridade e renda. Verificou-se, após a realização do *post-hoc*, que pais com nível superior apresentaram melhores resultados quanto ao comportamento no uso de antibióticos, quando comparados àqueles com nível médio (p=0,016). Na análise de associação com renda, verificou-se que pais com renda familiar superior a três SM apresentaram escores mais satisfatórios em relação aos que tinham renda de até um SM (p=0,002) ou renda entre um e dois SM (p=0,048).

No fator 4, Adesão ao tratamento, assim como no fator 2, observou-se presença de diferença de medianas nos escores da PAPA-Br entre grupos nas variáveis escolaridade e renda. No entanto, após a realização do *post-hoc* de Dunn, com valor de p ajustado, verificou-se diferença estatística somente para a variável renda, sendo que pais com renda superior a três SM apresentaram melhor adesão ao tratamento, quando comparados àqueles com renda de até um SM (p=0,007).

O fator 5, Conscientização dos pais sobre resistência aos antibióticos, mostrou diferenças significativas nas medianas entre grupos nas variáveis faixa etária, ocupação e renda. Após a realização do post-hoc de Dunn, identificou-se que: pais com idade entre 30 e 39 anos se apresentaram mais conscientes quanto à resistência de antibióticos, quando comparados aos que possuíam faixa etária de 19 a 29 anos (p=0,015); pais que eram profissionais de saúde obtiveram melhores escores neste fator, quando comparados àqueles que desemprenhavam outras funções (exceto, profissional de educação, dona de casa e autônomo) (p=0,023).

Uma análise de correlação entre as variáveis idade dos pais, renda e idade dos filhos também foi realizada, conforme apresentado na Tabela 3.

**Tabela 3:** Análise de correlação de Spearman das características sociodemográficas de pais e filhos com fatores da Escala de Percepção Parental sobre Antibióticos (N=209), Fortaleza, Ceará, Brasil, 2022

Fator	Idade dos pais		Renda		Idade das crianças	
	*r_s_*	p	r_s_	p	r_s_	p
Fator 1	- 0,084	0,226	-0,328	<0,001	0,086	0,216
Fator 2	- 0,035	0,617	-0,266	<0,001	0,078	0,261
Fator 4	0,005	0,938	-0,261	<0,001	-0,049	0,478
Fator 5	0,202	0,003	0,292	<0,001	0,012	0,868

*fator 1 - Conhecimento e crenças; fator 2 - Comportamentos; fator 4 - Adesão; fator 5 - Conscientização sobre resistência aos antibióticos; r_s_ - Coeficiente de correlação de Sperman; p - valor de significância do teste.*

A correlação entre a idade dos pais e o fator 5 foi estatisticamente significativa (n=209; r_s_ = 0,202; p=0,003), sugerindo que a conscientização sobre a resistência aos antibióticos tende a aumentar com a idade dos pais. A variável renda demonstrou correlações estatisticamente significativas com os seguintes fatores: fator 1 (n=209; r_s_ = -0,328; p < 0,001), indicando que à medida que a renda diminui, o conhecimento sobre antibióticos diminui; fator 2 (n=209; r_s_ = -0,266; p < 0,001), sugerindo que à medida que a renda diminui, os comportamentos relacionados aos antibióticos podem se tornar menos favoráveis; fator 4 (n=209; r_s_ = -0,261; p < 0,001), sugerindo que à medida que a renda diminui, a adesão ao tratamento com antibióticos pode diminuir; e fator 5 (n=209; r_s_ = 0,292; p < 0,001), indicando que à medida que a renda aumenta, a conscientização sobre a resistência aos antibióticos tende a aumentar. No entanto, é importante destacar que, embora estatisticamente significativas, a força das correlações foi fraca.

## DISCUSSÃO

A percepção dos pais sobre o uso de antibióticos em seus filhos tem sido amplamente investigada devido à relevância dos efeitos do uso indiscriminado desses medicamentos na saúde infantil. A avaliação realizada com a PAPA-Br, aliada à análise de dados sociodemográficos e clínicos, demonstrou consonância com os achados de estudos internacionais.

A predominância das mães entre os participantes é compatível com outros estudos que investigaram o conhecimento e as percepções dos pais sobre o uso de antibióticos em crianças, nos quais as mães constituíram a maior parte da amostra^(19-21)^. Esse resultado pode estar relacionado a normas culturais que frequentemente atribuem às mulheres o papel principal no cuidado dos filhos^(22)^. Da mesma forma, a média de idade das crianças é semelhante aos dados de estudo realizado em comunidades rurais do Peru^(23)^, reforçando a importância de investigações que considerem variáveis etárias no uso de antibióticos.

Neste estudo, o uso do medicamento ocorreu, principalmente, devido a infecções respiratórias. Resultados semelhantes foram encontrados em estudo realizado na Austrália, em que os pais relataram que os antibióticos são indicados para resfriados e gripe. Verificou-se, ainda, que pais com essa crença eram mais propensos a ter administrado antibióticos em seus filhos nos 12 meses anteriores à pesquisa^(24)^. Durante a etapa de tradução e adaptação transcultural da PAPA *Scale* para o português do Brasil, os pais também relataram que inflamação na garganta, gripe, pneumonia e bronquite foram as principais causas do uso de antibióticos por suas crianças^(18)^.

Observou-se a crença equivocada de que os antibióticos são eficazes no tratamento de qualquer tipo de infecção, inclusive as virais. Esse equívoco pode levar ao uso inadequado de antibióticos, contribuindo para a resistência bacteriana, um grave problema de saúde pública. Estudos apontam a relação direta entre resistência bacteriana e fatores como prescrição inadequada e uso incorreto de antibióticos^(25,26)^.

Essa tendência também foi encontrada na Turquia, onde uma proporção significativa de pais acreditava que os antibióticos eram eficazes tanto no tratamento de infecções bacterianas quanto no tratamento de infecções virais^(27)^. Tais percepções incorretas agravam o uso inadequado, especialmente quando associadas à prática, ainda frequente, da automedicação. Essa situação tem sido frequentemente observada em países com menor controle sobre a comercialização desses medicamentos, podendo trazer consequências negativas para crianças em desenvolvimento, como aumento da suscetibilidade a infecções^(9,28)^.

Em contrapartida, estudo realizado no Reino Unido identificou que as mães buscavam prescrição médica antes de administrar antibióticos aos filhos^(29)^. Na Suécia, a aquisição de antibióticos sem receita médica é baixa, refletindo maior conscientização pública sobre resistência bacteriana e um controle mais rigoroso na venda desses medicamentos^(30)^. Presume-se que essa diferença seja atribuída a políticas públicas mais eficazes em países desenvolvidos.

Quanto às fontes de informação mais citadas pelos pais sobre o uso de antibióticos, estão os profissionais de saúde e suas próprias experiências anteriores. Estudo qualitativo em Zomba, Malawi, com mães de baixa renda revelou que os profissionais de saúde são as principais fontes de informação sobre antibióticos, embora algumas mães também mencionem amigos, parentes, escolas e mídia^(31)^. Esse padrão foi observado em estudo no Líbano, onde a maioria dos participantes relatou adquirir conhecimento sobre saúde por meio de experiências prévias^(20)^.

Apesar do crescente uso da internet como fonte de informação, observa-se que, muitas vezes, o conteúdo acessado carece de embasamento técnico, sendo, por vezes, veiculado por redes sociais e influenciadores sem formação na área da saúde. Tal cenário contribui para a desinformação e reforça a necessidade de ações adequadas, especialmente em ambientes comunitários e escolares^(32-34)^.

A escolaridade demonstrou ser um fator fortemente associado à percepção e ao comportamento dos pais em relação ao uso de antibióticos. Estudos indicam que a probabilidade de uso de antibióticos é maior entre mães com menor escolaridade, enquanto práticas mais adequadas são observadas entre pais com ensino superior e instrução em saúde^(20,35)^. Além disso, pais com compreensão limitada sobre o funcionamento dos antibióticos frequentemente apresentam adesão insuficiente ao tratamento prescrito e descarte inadequado dos medicamentos, mesmo após orientações médicas^(36)^.

Em Bangladesh, foi identificado que variáveis como idade, gênero, nível educacional, situação de emprego e renda familiar estão associadas ao uso inapropriado de antibióticos. Esses achados evidenciam a importância de iniciativas educacionais voltadas ao uso responsável de antibióticos e à redução da resistência antimicrobiana^(37,38)^. De maneira semelhante, pais com menor nível educacional podem apresentar compreensão limitada sobre os potenciais danos e efeitos a longo prazo associados ao uso inadequado de antibióticos^(20,35,39)^.

Um comportamento preocupante relatado por alguns participantes foi o armazenamento de sobras de antibióticos para uso futuro, sugerindo equívocos sobre o reaproveitamento de medicamentos prescritos. Em Xangai, estudo revelou que a automedicação com antibióticos estocados é comum entre os pais^(40)^.

Esses achados ressaltam que os profissionais de saúde têm papel crucial no processo de sensibilização dos pais sobre a importância do uso adequado desses medicamentos. Intervenções educativas bem estruturadas e direcionadas, em conjunto com campanhas na televisão e mídias sociais, podem ajudar a reduzir a tendência à automedicação e a pressão dos pais sobre os profissionais de saúde para prescrever antibióticos quando seus filhos estão doentes^(21)^.

Nesse contexto, o enfermeiro exerce papel estratégico na promoção do uso racional de medicamentos em diferentes níveis de atenção à saúde. Na atenção primária, atua como agente educativo, utilizando linguagem acessível e recursos didáticos em ações individuais ou coletivas. Quando alinhadas ao contexto sociocultural e econômico das comunidades, intervenções como rodas de conversa, dramatizações, vídeos e jogos se tornam mais efetivas e acessíveis.

Já nos contextos hospitalar e domiciliar, o profissional contribui para a continuidade segura do cuidado ao orientar pais e responsáveis no momento da alta e no acompanhamento em casa. Ao sensibilizar sobre os riscos da automedicação e da interrupção precoce do tratamento, o enfermeiro fortalece a adesão terapêutica e atua na prevenção de eventos adversos^(41)^. Assim, reforça-se a importância das ações educativas conduzidas por enfermeiros integradas às diversas fases do cuidado e adaptadas às realidades dos pacientes e suas famílias.

### Limitações do estudo

O presente estudo possui algumas limitações, como a restrição da coleta apenas à capital do Ceará, o que impede a comparação com populações de outras regiões do Brasil. Além disso, o método de recrutamento usado, bola de neve, pode ter introduzido viés de seleção, limitando a diversidade da amostra. Essa abordagem pode levar a uma amostra menos representativa da população geral, o que pode impactar os achados. Estudos futuros devem considerar a inclusão de amostras de diversos contextos regionais para avaliar a consistência dos resultados.

### Contribuições para as áreas da saúde e da enfermagem

Os pontos fortes deste estudo incluem o fato de ser o primeiro a utilizar a PAPA-Br, uma versão adaptada culturalmente ao Brasil, que permitiu uma análise das percepções dos pais brasileiros sobre o uso de antibióticos. Aos considerar fatores culturais específicos que podem influenciar o uso desses medicamentos, este trabalho oferece uma contribuição inédita e relevante para o campo. Além disso, a coleta de dados em uma capital do nordeste brasileiro amplia o impacto do estudo, fornecendo *insights* valiosos sobre uma região frequentemente sub-representada em pesquisas científicas, o que fortalece a base para o desenvolvimento de futuras intervenções educacionais adaptadas às necessidades locais.

A identificação dos fatores que influenciam os pais no uso inadequado ou incorreto dos antibióticos pode auxiliar no desenvolvimento de estratégias educativas específicas que melhorem o conhecimento, as crenças, os comportamentos, a adesão e a conscientização dos pais. Essas estratégias podem, ainda, facilitar uma comunicação eficaz entre os profissionais de saúde e os pais, contribuindo para uma terapia mais eficaz e para a administração segura de antibióticos, com impactos positivos tanto na saúde da criança quanto na redução da resistência antimicrobiana.

No contexto da prática de enfermagem, os resultados do estudo fornecem subsídios para a atuação do enfermeiro no desenvolvimento e implementação de estratégias educativas direcionadas às necessidades da população, especialmente nos serviços de atenção primária. O profissional pode utilizar os achados para estruturar ações educativas voltadas aos cuidadores, com foco na promoção do uso racional de antibióticos, esclarecimento sobre a diferença entre infecções virais e bacterianas, e sensibilização sobre os riscos do uso indevido desses medicamentos. Além disso, o enfermeiro pode atuar junto às equipes multiprofissionais na implementação de protocolos de prescrição segura e no acompanhamento da adesão ao tratamento. Dessa forma, o estudo contribui para fortalecer a prática baseada em evidências e o papel da enfermagem na prevenção da resistência antimicrobiana.

## CONCLUSÕES

Os resultados evidenciaram que as decisões dos pais sobre o uso de antibióticos por seus filhos no domicílio são impactadas por suas crenças, práticas baseadas em experiências prévias e pelo nível de escolaridade, destacando a necessidade de intervenções direcionadas. A pesquisa contribui para a saúde e a sociedade, ao reforçar a importância de estratégias educativas que promovam o conhecimento e comportamentos adequados, além de fortalecer a comunicação entre pais e profissionais de saúde. Essas medidas são essenciais para garantir o uso racional de antibióticos, minimizando riscos à saúde das crianças e combatendo a resistência antimicrobiana.

## Data Availability

Os dados da pesquisa estão disponíveis no corpo do artigo.
